# A Comparison between the Effects of Glucantime, Topical Trichloroacetic Acid 50% plus Glucantime, and Fractional Carbon Dioxide Laser plus Glucantime on Cutaneous Leishmaniasis Lesions

**DOI:** 10.1155/2016/6462804

**Published:** 2016-04-11

**Authors:** Fariba Jaffary, Mohammad Ali Nilforoushzadeh, Amirhossein Siadat, Elaheh Haftbaradaran, Nazli Ansari, Elham Ahmadi

**Affiliations:** ^1^Skin Diseases and Leishmaniasis Research Center, Isfahan University of Medical Sciences, Isfahan 8187698191, Iran; ^2^Skin and Stem Cell Research Center, Tehran University of Medical Sciences, Tehran 1937957514, Iran; ^3^Department of Dermatology, Isfahan University of Medical Sciences, Isfahan 8174675731, Iran

## Abstract

*Background.* Cutaneous leishmaniasis is an endemic disease in Iran. Pentavalent antimonial drugs have been the first line of therapy in cutaneous leishmaniasis for many years. However, the cure rate of these agents is still not favorable. This study was carried out to compare the efficacies of intralesional glucantime with topical trichloroacetic acid 50% (TCA 50%) + glucantime and fractional carbon dioxide laser + glucantime in the treatment of cutaneous leishmaniasis.* Methods.* A total of 90 patients were randomly divided into three groups of 30 to be treated with intralesional injection of glucantime, a combination of topical TCA 50% and glucantime, or a combination of fractional laser and glucantime. The overall clinical improvement and changes in sizes of lesions and scars were assessed and compared among three groups.* Results.* The mean duration of treatment was 6.1 ± 2.1 weeks in all patients (range: 2–12 weeks) and 6.8 ± 1.7, 5.2 ± 1.0, and 6.3 ± 3.0 weeks in glucantime, topical TCA plus glucantime, and fractional laser plus glucantime groups, respectively (*P* = 0.011). Complete improvement was observed in 10 (38.5%), 27 (90%), and 20 (87%) patients of glucantime, glucantime + TCA, and glucantime + laser groups, respectively (*P* < 0.001).* Conclusion.* Compared to glucantime alone, the combination of intralesional glucantime and TCA 50% or fractional CO_2_ laser had significantly higher and faster cure rate in patients with cutaneous leishmaniasis.

## 1. Introduction

Cutaneous leishmaniasis is an endemic disease in tropical areas with 90% of cases emerging from Iran, Afghanistan, Pakistan, Iraq, Saudi Arabia, Brazil, and Peru [1]. The infection rate reaches 70% in some hyperendemic foci in Iran with 10–20 thousand new cases annually [2]. The disease imposes a large burden on patient's physical, psychological, social, and economic well-being [3].

*Leishmania major* and* Leishmania tropica* are the main parasite species responsible for cutaneous leishmaniasis in the Old World. The sandflies* Phlebotomus sergenti* and* Phlebotomus papatasi* are the main vector of the Old World's cutaneous leishmaniasis.* Rhombomys opimus* serves as the main reservoir of the disease [4].

The cutaneous lesion usually develops on the exposed parts of the body within a few weeks to several months of the sandfly bite, presenting as an erythematous papule which gradually enlarges in size to become a nodule. Eventually the lesion becomes crusted with raised indurated borders. There may be multiple lesions. Spontaneous resolution of lesion may occur over months to years, often leaving a disfiguring atrophic scar. The lesion is usually painless unless it becomes secondarily infected with bacteria or fungi [5].

Although most cases of cutaneous leishmaniasis heal spontaneously, different drugs for systemic and topical treatment have been introduced for those with disfiguring or relapsing lesions. Pentavalent antimony compounds are the most common treatment for cutaneous leishmaniasis. The failure rate with regular use of these compounds is about 10–15% [6]. The exact mechanism of action of antimony compounds is still obscure. Pentavalent antimonials may inhibit the parasite's glycolytic and fatty acid oxidative activity resulting in diminished synthesis of adenosine triphosphate (ATP) and guanosine triphosphate (GTP). The process would eventually lead to the death of parasite in amastigote stage of the life cycle. It is also suggested that T cell activation is required for the action of pentavalent antimonials and miltefosine [7, 8]. The adverse effects of the drug and difficult administration are the main limiting factor for use of these compounds.

Trichloroacetic acid (TCA) peeling is a traditional and popular method for skin rejuvenation. This chemical peeling method has been widely utilized in the management of other aesthetic dermatologies including actinic keratoses, scars, and mottled pigmentation [9]. TCA is usually used as a superficial peeling agent; however, higher TCA concentrations will penetrate to the mid-reticular dermis [10]. TCA peeling induces the skin stress response system resulting in reconstitution of the epidermis and dermis through wound healing processes [11]. This ability makes the TCA peeling a potential therapeutic option for cutaneous leishmaniasis [12, 13].

Laser technology is already an established treatment for a wide variety of dermatological problems. Recent innovations in fractional photothermolysis have allowed for the achievement of the desired aesthetic results with minimum pain and pigmentary changes [14]. Fractional CO_2_ laser alone has shown promising results against cutaneous leishmaniasis [15–17]. However, clinical data are still lacking on the efficacy of photothermolysis compared to other traditional treatments. To the best of our knowledge, no previous trial has evaluated the efficacy of fractional CO_2_ laser combined with intralesional glucantime. Therefore, this study was undertaken to compare the efficacy and safety of traditional glucantime with its combination with TCA 50% and fractional laser in the treatment of cutaneous leishmaniasis.

## 2. Materials and Methods

### 2.1. Study Population

This prospective interventional randomized controlled study was conducted in the Skin Disease and Leishmaniasis Research Center of Isfahan University of Medical Sciences, Isfahan, Iran, between April 2010 and May 2011. The study was approved by our Institutional Research Ethics Board and written informed consent was obtained from all patients.

Patients older than 5 years with biopsy-confirmed cutaneous leishmaniasis were eligible if they had the following criteria: lesion diameter < 3 cm, disease duration < 12 weeks, lesion-to-eyelid distance > 2 cm, and no history of systemic or topical therapy for cutaneous leishmaniasis. Patients with any of the following criteria were excluded: pregnancy, lactation, immunosuppressive therapy, and serious side effects of medication.

Eligible patients were then randomly assigned by a computer-generated list to receive either glucantime (group A) or glucantime plus topical trichloroacetic acid 50% (group B) or glucantime plus fractional CO_2_ laser (group C) as described below.

### 2.2. Treatment

Group A: Intralesional injection of meglumine antimoniate (glucantime; Specia, France) was performed until complete blanching of the lesion and its margins. The procedure was repeated twice a week for a maximum of 8 weeks or until complete healing of the lesion.

Group B: After intralesional injection of glucantime, topical trichloroacetic acid 50% (Merck, Berlin, Germany) was applied to the lesion by a cotton applicator. When the lesion was frosted, the acid was neutralized by water and the scar site was covered with Vaseline. The procedure was repeated for glucantime (twice a week) and TCA 50% (once a week) for up to 8 weeks. Group C: After intralesional injection of glucantime, fractional CO_2_ laser (Qray FRX, DOSIS M&M, South Korea) with the following parameters was used: energy, 25 J, dot cycle, 5, pixel pitch, 1 mm, and 1 pass. Patients in this group received a total of 2 laser treatment sessions at 2-week intervals with repeated intralesional injection of glucantime twice a week for up to 8 weeks.

### 2.3. Outcome Measures

Patients in all three groups were followed up weekly for the next 6 months. Any side effect or drug-related complications were recorded. Standard digital photographs using identical camera settings, lighting, and patient positioning were taken at baseline and in the following visits. The clinical response was rated as complete improvement (complete reepithelialization of the lesion with negative direct skin smear result), partial improvement (50–75% improvement of the lesion size), and no change in the lesion appearance. The improvement of scar was also evaluated according to the patient's satisfaction, the morphology of the lesion, the level of induration, and the level of atrophy and scored as follows: score of 1 (less than 25% improvement), score of 2 (25% to 50% improvement), score of 3 (51% to 75% improvement), and score of 4 (76% to 100% improvement).

### 2.4. Statistical Analysis

Statistical analysis was performed using SPSS version 19 software (SPSS Inc., Chicago, IL, USA). To compare baseline and outcome measures among groups, chi-square and ANOVA tests were used. Results are expressed as mean ± standard deviation (SD) or numbers (percentages). *P* value less than 0.05 was considered significant.

## 3. Results

There were 90 patients (30 in each group) with the mean age of 24.5 ± 14 years. A total of 76 patients (84.5%) completed treatment and were followed up for 60 days. There were 53 patients with a single lesion, 29 with double lesions, and 8 with triple lesions. The most common lesion types were nodule (48.9%) and plaques (35.6%). The three groups were similar in terms of age, sex, and number and location of lesions (*P* > 0.05). The most common lesion sites were the upper and lower extremities (Table 1).

Table 2 shows the rates of improvement until the last follow-up. Complete improvement was observed in 10 (38.5%), 27 (90%), and 20 (87%) patients of glucantime, glucantime + TCA, and glucantime + laser groups, respectively (*P* < 0.001). The average healing times were 6.8 ± 1.7 weeks in the glucantime group, 5.2 ± 1 weeks in the glucantime + TCA group, and 6.3 ± 3 weeks in the glucantime + CO_2_ laser group (*P* = 0.011).

**Figure 1:**
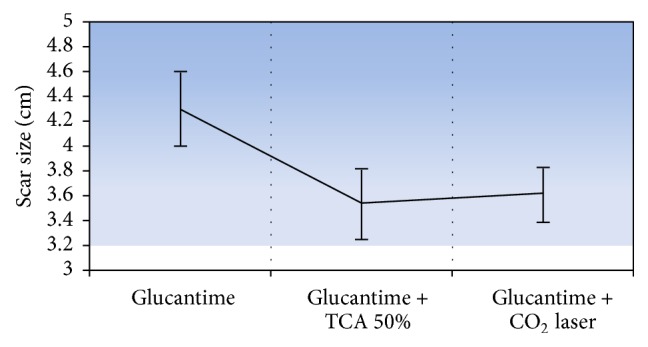
The mean and 95% CI of scar size in three groups.

The scar score was significantly lower in the glucantime + TCA and glucantime + fractional laser groups as compared with the glucantime group. No statistically significant difference of scar score was noted between the TCA and fractional laser groups (*P* = 0.9) (Figure 1).

No serious side effects or complications were encountered during the study period. Local hypersensitivity was observed in five patients while two patients experienced secondary bacterial infection which responded to topical antibiotics. Satellite nodules were found in three patients and one patient experienced enlargement of the lesion size which all healed spontaneously. No recurrence of the lesions was noted. The side effects were similar in three groups.

## 4. Discussion

Endemic cutaneous leishmaniasis in Iran has a huge social and economic burden [18, 19]. Different treatment strategies have been described and many have been clinically proven to be effective against cutaneous leishmaniasis. The choice of treatment is often determined by the characteristics of the patient (age, gender, location of lesion, and immunity) and parasite (species variation). The treatment efficacy is also dependent on the geographical area [20]. Glucantime remains the first-line treatment for cutaneous leishmaniasis despite the widespread drug resistance and high incidence of side effects [21]. However, over the past decade, multiple clinical trials have been performed to substitute pentavalent antimony compounds.

Our current results indicate that the combined treatment of glucantime with TCA 50% and fractional CO_2_ laser is much more effective than glucantime alone. On the other hand, combined therapy was associated with shorter healing duration. No significant difference between TCA 50% and fractional CO_2_ laser regarding the overall improvement and side effects was encountered. Additionally, assessment of cutaneous leishmaniasis scar revealed greater response to TCA and fractional CO_2_ laser both objectively and subjectively.

Previous investigations reported the TCA effectiveness of 48–100% [22, 23]. TCA could be administered easily anywhere on the body and the procedure is safe and efficacious in experienced hands. Although TCA is more available than fractional photothermolysis, often multiple treatments over several sessions are required to achieve the desired outcome.

The results of our laser therapy were in accordance with previous reports. In a study by Asilian et al. [24] continuous CO_2_ laser was superior to glucantime in the treatment of cutaneous leishmaniasis in terms of efficacy (93.7% versus 83.3%, *P* < 0.0007), healing time (1 month versus 3 months), and side effects (4.5% versus 24%). In another study the cure rates of CO_2_ laser and glucantime + cryotherapy were reported as 93.7 and 73%, respectively. However the side effects were similar in two groups [25]. No recurrences have been reported after CO_2_ laser therapy in several years of follow-up [26]. In comparison with our previous survey [22] reporting 44.8% of improvement with CO_2_ laser, here we achieved a double rate of improvement. As far as we know our current investigation is the first randomized controlled trial using fractional CO_2_ laser in the treatment of cutaneous leishmaniasis. Higher success rate and lower complications observed here are probably due to the nature of fractional technology. However, more well-designed clinical trials are needed to confirm the potential benefits of this new technology.

## 5. Conclusions

Combination therapy of intralesional glucantime with TCA 50% or fractional CO_2_ laser is much more effective than intralesional glucantime alone in the treatment of cutaneous leishmaniasis. Additionally, combination therapy accelerates the healing process without any serious side effects.

## Figures and Tables

**Table 1:** Distribution of scar sites.

Lesion site	Number of lesions
Upper extremities	43
Lower extremities	15
Face	8
Trunk	5
Upper extremities + trunk	4
Upper extremities + neck	4
Neck	4
Upper extremities + lower extremities	4
Upper extremities + face	2
External ear	1

**Table 2:** The overall clinical improvement.

Clinical improvement, *n* (%)	Group A (glucantime)	Group B (glucantime + TCA 50%)	Group C (glucantime + CO_2_ laser)	*P* value
Complete	10 (38.5%)	27 (90%)	20 (87%)	0.001
Partial	14 (53.8%)	3 (10%)	0	
No change	2 (7.7%)	0	0	

## References

[1] Choi C. M., Lerner E. A. (2001). Leishmaniasis as an emerging infection. *Journal of Investigative Dermatology Symposium Proceedings*.

[2] Nilforoushzadeh M. A., Jaffary F., Moradi S., Derakhshan R., Haftbaradaran E. (2007). Effect of topical honey application along with intralesional injection of glucantime in the treatment of cutaneous leishmaniasis. *BMC Complementary and Alternative Medicine*.

[3] Kassi M., Afghan A. K., Rehman R., Kasi P. M., Kassi M. (2008). Marring leishmaniasis: the stigmatization and the impact of cutaneous leishmaniasis in Pakistan and Afghanistan. *PLoS Neglected Tropical Diseases*.

[4] Reithinger R., Dujardin J.-C., Louzir H., Pirmez C., Alexander B., Brooker S. (2007). Cutaneous leishmaniasis. *The Lancet Infectious Diseases*.

[5] Piscopo T. V., Mallia Azzopardi C. (2007). Leishmaniasis. *Postgraduate Medical Journal*.

[6] Dowlati Y. (1996). Cutaneous leishmaniasis: clinical aspect. *Clinics in Dermatology*.

[7] Arevalo I., Ward B., Miller R. (2001). Successful treatment of drug-resistant cutaneous leishmaniasis in humans by use of imiquimod, an immunomodulator. *Clinical Infectious Diseases*.

[8] Ghosh M., Roy K., Roy S. (2013). Immunomodulatory effects of antileishmanial drugs. *Journal of Antimicrobial Chemotherapy*.

[9] Committee for Guidelines of Care for Chemical Peeling (2012). Guidelines for chemical peeling in Japan (3rd edition). *The Journal of Dermatology*.

[10] Nguyen T. H., Rooney J. A. (2000). Trichloroacetic acid peels. *Dermatologic Therapy*.

[11] Kimura A., Kanazawa N., Li H.-J., Yonei N., Yamamoto Y., Furukawa F. (2012). Influence of chemical peeling on the skin stress response system. *Experimental Dermatology*.

[12] Siadat A., Jaffary F., Nilforoushzadeh M., Ansari N., Moradi S. (2011). The comparison between trichloroacetic acid 50% and CO_2_ laser in the treatment of cutaneous leishmaniasis scar. *Indian Journal of Dermatology*.

[13] Nilforoushzadeh M. A., Fatemi Naieni F., Sattar N., Haftbaradaran E., Jaffary F., Askari G. H. (2012). The efficacy of intralesional meglumine antimoniate (glucantime) versus a combination of topical trichloroacetic acid 50% and local heat therapy by non-ablative radiofrequency on cutaneous leishmaniasis lesions. *Journal of Research in Medical Sciences*.

[14] Saedi N., Jalian H. R., Petelin A., Zachary C. (2012). Fractionation: past, present, future. *Seminars in Cutaneous Medicine and Surgery*.

[15] Lapidoth M., Halachmi S., Cohen S., Amitai D. B. (2014). Fractional CO_2_ laser in the treatment of facial scars in children. *Lasers in Medical Science*.

[16] Alghamdi K. M. (2010). Successful treatment of atrophic scars from cutaneous leishmaniasis using a fractional laser. *Journal of Cutaneous Medicine and Surgery*.

[17] Nilforoushzadeh M. A., Minaravesh S. H., Jaffary F., Siadat A. H., Haftbaradaran E. (2014). Comparison the efficacy of ablative CO_2_ laser and fractional CO_2_ laser on the healing of cutaneous leishmaniasis scars. *Advanced Biomedical Research*.

[18] Alvar J., Vélez I. D., Bern C. (2012). Leishmaniasis worldwide and global estimates of its incidence. *PLoS ONE*.

[19] Karami M., Doudi M., Setorki M. (2013). Assessing epidemiology of cutaneous leishmaniasis in Isfahan, Iran. *Journal of Vector Borne Diseases*.

[20] González U., Pinart M., Reveiz L., Alvar J. (2008). Interventions for old world cutaneous leishmaniasis. *Cochrane Database of Systematic Reviews*.

[21] Hadighi R., Mohebali M., Boucher P., Hajjaran H., Khamesipour A., Ouellette M. (2006). Unresponsiveness to Glucantime treatment in Iranian cutaneous leishmaniasis due to drug-resistant *Leishmania tropica* parasites. *PLoS Medicine*.

[22] Nilforoushzadeh M. A., Jaffary F., Ansari N., Moradi S., Siadat A. H. (2011). The comparison between trichloroacetic acid 50% and CO_2_ laser in the treatment of cutaneous leishmaniasis scar. *Indian Journal of Dermatology*.

[23] Ali N. M., Fariba J., Elaheh H., Ali N. (2012). The efficacy of 5% trichloroacetic acid cream in the treatment of cutaneous leishmaniasis lesions. *Journal of Dermatological Treatment*.

[24] Asilian A., Sharif A., Faghihi G., Enshaeieh S., Shariati F., Siadat A. H. (2004). Evaluation of CO_2_ laser efficacy in the treatment of cutaneous leishmaniasis. *International Journal of Dermatology*.

[25] Shamsi Meymandi S., Zandi S., Aghaie H., Heshmatkhah A. (2011). Efficacy of CO_2_ laser for treatment of anthroponotic cutaneous leishmaniasis, compared with combination of cryotherapy and intralesional meglumine antimoniate. *Journal of the European Academy of Dermatology and Venereology*.

[26] Babajev K. B., Babajev O. G., Korepanov V. I. (1991). Treatment of cutaneous leishmaniasis using a carbon dioxide laser. *Bulletin of the World Health Organization*.

